# Nursing managers’ perspectives on factors contributing to abuse in psychiatric hospitals: A reflective thematic analysis

**DOI:** 10.1016/j.ijnsa.2025.100388

**Published:** 2025-08-19

**Authors:** Kei Matoba, So Yayama, Taiki Teshima, Akiko Miki

**Affiliations:** Faculty of Nursing, Kansai Medical University, Hirakata, Osaka, Japan

**Keywords:** Mentally ill persons, Nurse administrators, Patient abuse, Psychiatric nursing, Victimization, Violence

## Abstract

**Background:**

Research has identified evidence of coercion and abuse in psychiatric hospitals. The psychiatric care system in Japan is characterized by a custodial approach and long-term inpatient care, potentially increasing the risk of abuse. However, few studies have examined the occurrence and dynamics of abuse in psychiatric institutions, and there is a major research gap regarding the roles of hospital culture and management in contributing to inpatient abuse.

**Objective:**

To investigate the experiences of psychiatric nursing managers of the factors contributing to inpatient abuse in psychiatric hospitals.

**Design:**

A multisite, phenomenological, qualitative, and descriptive study.

**Settings:**

Four hospitals in Japan that were either psychiatric hospitals or general hospitals dominated by psychiatric wards.

**Participants:**

Eighteen nurse managers (including directors of nursing, deputy directors of nursing, and head nurses) working in psychiatric hospitals or in general hospitals dominated by psychiatric wards.

**Methods:**

From May 2023 to July 2023, individual semi-structured interviews were conducted with each participant to obtain data on factors contributing to abuse in psychiatric hospitals. Reflexive thematic analysis was used to analyze the data. The data were coded, and the codes organized into themes and subthemes.

**Results:**

Six themes and 23 subthemes were extracted that described participants’ perspectives on systemic, personal, and environmental contributors to inpatient abuse. The themes were ‘Structural challenges embedded in psychiatric care systems’ (e.g., finances, staffing, working conditions); ‘Organizational cultures lacking self-correction’ (e.g., the insularity of psychiatric hospitals, use of outdated nursing practices); ‘Dysfunctional team dynamics that undermine professional competence’ (e.g., suppression of nurses’ opinions and autonomy); ‘Asymmetries in patient–nurse relationships’ (e.g., power imbalances); ‘Illness factors that complicate the detection of abuse’ (e.g., interpretation of abuse as symptoms); and ‘The fragility of psychiatric nurses’ professional identity’ (e.g., lack of skills, experience, confidence, and fulfillment).

**Conclusions:**

The results show that nurse managers play a key role in identifying institutional, personal, and relationship-based factors that contribute to inpatient abuse in psychiatric wards. The findings indicate a need for more policies to support patient-centered care and develop the role of nurse managers to create safer psychiatric wards.

**Study registration:**

Not registered.

**Tweetable abstract:**

nurse managers’ perspectives on contributors to inpatient abuse in psychiatric hospitals

**What is already known**

• People with mental illness are vulnerable to abuse, even within psychiatric hospitals.

• Abuse may occur through coercive practices embedded in psychiatric care settings.

**What this paper adds**

• Nursing managers in this study described systemic, cultural, and interpersonal factors that they perceived as contributing to abuse.

• These perspectives suggest that abuse may be shaped by staff characteristics, dysfunctional team dynamics, and challenges related to patient care.

• The study highlights the need for leadership-driven reforms to promote ethical care and improve institutional culture.

Alt-text: Unlabelled box

## 1 Background

Psychiatric hospitals are intended to provide a safe and therapeutic environment for people with mental illness. However, previous research suggests that these institutions may also be sites of abuse in which systemic and organizational factors contribute to patient harm (Robins et al., 2005; Rossa-Roccor et al., 2020). Although people with mental illness are often perceived as potential perpetrators of violence, they are also at high risk of victimization (Sariaslan et al., 2020). Recognizing the vulnerability of patients with psychiatric disorders, the World Health Organization has emphasized the need for a human rights-based approach to mental healthcare that highlights the importance of safeguarding patients from violence and coercion (World Health Organization & Office of the United Nations High Commissioner for Human Rights, 2023). However, despite such global recommendations, there is evidence that coercive practices remain prevalent in psychiatric hospitals, and sometimes lead to mistreatment by staff (Askew et al., 2019; Berzins et al., 2020).

Abuse in psychiatric hospitals encompasses physical, psychological, sexual, and economic harm, as well as neglect and violations of human rights. Much research has focused on the victimization of people with mental disorders in community settings (de Mooij et al., 2015; de Vries et al., 2019); fewer studies have examined the prevalence and dynamics of abuse in psychiatric institutions. However, previous studies indicate that 13%–16% of psychiatric inpatients report experiencing abuse by hospital staff, and such experiences vary according to gender and race (Cusack et al., 2007; Frueh et al., 2005; Rossa-Roccor et al., 2020). In addition to direct physical or psychological harm, psychiatric hospitals may inherently foster conditions that facilitate abuse. Institutional structures and care systems originally designed for patient protection can inadvertently cause distress or lead to mistreatment (Robins et al., 2005). Addressing these issues is essential to ensuring the dignity and safety of psychiatric inpatients.

In Japan, a series of abuse incidents in psychiatric hospitals between 2020 and 2023—including cases involving nursing staff—sparked public outcry and led to legal reforms aimed at improving mental healthcare and preventing abuse. However, systemic challenges remain. The psychiatric hospital system in Japan has long been characterized by high psychiatric bed numbers, prolonged inpatient stays, and an aging inpatient population (Ministry of Health, Labour and Welfare, 2022; Okayama et al., 2020). Psychiatric care across Asia has predominantly been shaped by the biomedical model, and emphasizes a custodial approach to treatment that prioritizes control and institutionalization over patient autonomy (Kuek et al., 2020). This managerial approach in psychiatric hospitals can be associated with restrictions or violations of patients’ human rights, thereby increasing the risk of abuse (Shiina et al., 2018). Although nursing managers play an essential role in fostering ethical and safe care environments (McGarry, 2019), research on the managerial and organizational factors that contribute to the abuse of psychiatric inpatients remains scarce. Most studies have focused on patient experiences of coercion and mistreatment, so there are substantial gaps in understanding how hospital management and institutional culture influence the occurrence of abuse, particularly in relation to psychiatric nursing leadership. This study aimed to address this gap by examining the perspectives of nursing managers on the factors that contribute to abuse in psychiatric hospitals. The goal was to help inform targeted interventions to improve patient safety and ethical care practices. To achieve this, we used Braun and Clarke’s thematic analysis approach (Braun and Clarke, 2021), which enables the identification of latent patterns within qualitative data. This framework allowed us to explore the underlying systemic and cultural factors that contribute to abuse in psychiatric hospitals, providing a deeper understanding of the institutional mechanisms that perpetuate mistreatment.

## 2 Methods

### 2.1 Study design

To identify the factors that contribute to patient abuse in psychiatric hospitals as experienced by nursing managers, we conducted a phenomenological study using a multisite, qualitative, descriptive design. Data were collected through individual semi-structured interviews. By including nursing managers from diverse settings, we aimed to gain a broader understanding of the factors that contribute to abuse from the varied experiences and perspectives of the participants. To systematically explore the complex patterns of factors contributing to abuse that emerged, we used reflexive thematic analysis (RTA). RTA was chosen over other forms of thematic analysis because it allows for a flexible, interpretative approach that integrates researcher reflexivity into the analytical process. Unlike coding reliability or codebook thematic analysis, RTA does not rely on predefined themes or rigid coding structures; rather, it enables researchers to actively engage with the data and construct themes dynamically based on emergent patterns (Braun and Clarke, 2006, 2019a). This approach is particularly suitable for the present data because it allowed us to explore the systemic and cultural factors influencing institutional mistreatment in psychiatric hospitals. Such factors are shaped by subjective experiences and organizational dynamics. Study registration was not applicable for this qualitative interview study, and the study was not registered.

### 2.2 Sample and setting

We used purposive sampling. Between May 2023 and July 2023, the first author sent an email to the nursing directors of seven psychiatric hospitals, explaining the aims and methods of the study and requesting their cooperation. We selected hospitals that were actively engaged in preventing abuse. Specifically, the selected hospitals had implemented strategies to address abuse incidents, minimized the use of coercive treatment (with some wards achieving zero coercive interventions), or were the main psychiatric institutions in their respective regions. We sent a document explaining the research to the nurse managers of hospitals that provided consent, and requested individual consent from those nurse managers who met the following inclusion criteria: (a) nurse managers working in general hospitals or psychiatric hospitals where psychiatric wards constitute more than half the beds, (b) nurse managers not working in outpatient departments, and (c) nursing managers holding positions such as director of nursing, deputy director of nursing, or head nurse of a ward. We obtained both written and verbal informed consent either in person or via an online video call. Participants were informed about the study purpose, methods, expected benefits, potential risks, the funding source, and their right to refuse participation. Following the interview, participants received a payment of 3,000 yen (approximately 20 US dollars).

A total of 18 participants from four hospitals were included. Three of the four hospitals were collaborating facilities used by researchers for educational purposes, and relationships with the nursing managers had been established before the study began. Eight participants were in general hospitals dominated by psychiatric wards and the other participants were in psychiatric hospitals. The four hospitals had between 300 and 900 psychiatric beds.

### 2.3 Data collection and processing

Each participant was interviewed once, resulting in a total of 18 interviews. The semi-structured interviews were conducted either face-to-face or via online video calls, depending on the participant’s preference by first and/or second author. The interviews took place in a private setting to ensure confidentiality, and lasted between 45 and 75 minutes, with an average of 54 minutes.

We used an interview guide comprising three key questions on the following topics: (1) examples of abuse in psychiatric hospitals and the factors contributing to their occurrence, (2) approaches and strategies for preventing abuse, and (3) challenges in preventing abuse. To elicit in-depth narratives, we sent the interview guide to the participants beforehand and asked them to consider their responses in advance. During the interviews, participants were asked to describe specific episodes related to abuse. As the data collection progressed, insights from earlier interviews informed follow-up questions in subsequent interviews to further explore emerging themes. However, these themes were not predefined before the study; rather, they evolved iteratively based on the data collected.

### 2.4 Data analysis

Data analysis followed Braun and Clarke’s six-step RTA framework (Braun and Clarke, 2006; Braun and Clarke, 2021). In Step 1, the researcher (K.M.) transcribed the data verbatim and critically reread the transcripts to gain familiarity with the content and acquire an overall understanding of the data. During this process, potential coding areas were highlighted and initial ideas were documented. In Step 2, the researcher (K.M.) descriptively coded the data in alignment with the research question. As part of the coding process, we iteratively refined the codes based on the data. New codes were created when novel patterns emerged, while existing codes were adjusted and refined to accommodate new data. This process was dynamic and reflexive, and ensured that the codes accurately captured the evolving themes within the dataset. In Steps 3 and 4, the researcher (K.M.) developed potential themes by reviewing the coded data and grouping codes with similar patterns. These themes were refined by relating them to both individual data segments and the whole dataset. Overlapping themes were merged, and when subthemes were identified, broader overarching themes were developed to ensure coherence and distinction between each theme and subtheme. In Step 5, all researchers discussed and refined the themes and subthemes to ensure their distinctiveness and establish clear relationships between them. Multiple rounds of coding, code categorization into themes, and iterative refinement of themes and subthemes were conducted before the final phase of analysis. In Step 6, we selected representative examples for each theme and subtheme, organized the data, and prepared the final report.

### 2.5 Rigor and trustworthiness

To ensure the rigor of the study, we applied the criteria of trustworthiness, credibility, dependability, and transferability (Lincoln and Guba, 1985). To increase credibility, we invited those participants who expressed a willingness to do so to confirm that the transcribed interviews accurately reflected their intended meaning. However, none of the participants requested to provide revisions. The analysis was collaboratively discussed among all authors, and feedback was incorporated to increase analytical depth and reflexivity. To ensure confirmability, we selected and presented narrative data that best represented the themes and subthemes. Throughout the analysis process, we referred to Braun and Clarke’s (Braun and Clarke, 2021) RTA checklist to ensure adherence to the methodological framework and maintain consistency in our approach. We used NVivo12 (Lumivero, https://lumivero.com/) to help with the analysis.

As RTA emphasizes the active role of the researcher in knowledge production (Braun and Clarke, 2019b), we engaged in continuous reflexive discussions to critically examine how our own backgrounds and perspectives influenced the analytical process. All authors had previous research experience in psychiatric hospital abuse and violence, which provided valuable insights that shaped the depth of inquiry in the interviews. The first author, who has a background in qualitative research methodology, provided a contrasting perspective that facilitated critical reflection on emerging themes and ensured a balance between depth of interpretation and methodological rigor. This iterative reflexive process ensured that our analysis remained transparent, consistent, and methodologically sound (Byrne, 2022).

### 2.6 Ethical approval and informed consent

The study was approved by the local Ethics Review Board. Prior to data collection, all participants were provided with a detailed explanation of the study’s purpose, content, and measures to ensure confidentiality. They were informed that participation was voluntary, that they could withdraw at any time, and that their anonymity would be protected. Written informed consent was obtained after participants demonstrated their willingness to consent. To maintain confidentiality, all data and quotes were pseudonymized. No adverse events occurred during the interviews.

## 3 Results

Eighteen nursing managers from four psychiatric hospitals participated in this study. Of these, thirteen were head nurses at wards (eight female), two were directors of nursing (one female), two were deputy directors of nursing (one female), and one was a nursing adviser (female). Participants had an average age of 53 years (range: 37–63 years) and an average managerial experience of 12 years (range: 3–25 years) (Table 1).

**Table 1:** Characteristics of the participants.

Participant	Age	Gender	Position	Managerial experience (years)
1	51	M	Deputy Director of Nursing	9
2	48	M	Head Nurse	5
3	48	F	Head Nurse	8
4	46	F	Head Nurse	6
5	60	F	Head Nurse	18
6	56	F	Head Nurse	9
7	47	F	Head Nurse	6
8	53	M	Director of Nursing	21
9	44	M	Head Nurse	11
10	63	F	Nursing Adviser	22
11	62	F	Head Nurse	18
12	37	M	Head Nurse	6
13	60	F	Head Nurse	22
14	63	M	Head Nurse	25
15	60	F	Head Nurse	9
16	48	F	Deputy Director of Nursing	3
17	59	F	Director of Nursing	20
18	53	M	Head Nurse	3

Six themes and 23 subthemes were developed to describe nursing managers’ perspectives on factors contributing to abuse against inpatients in psychiatric hospitals. The themes are as follows: ‘Structural challenges embedded in psychiatric care systems’, ‘Organizational cultures lacking self-correction’, ‘Dysfunctional team dynamics that undermine professional competence’, ‘Asymmetries in patient–nurse relationships’, ‘Illness factors that complicate the detection of abuse’, and ‘The fragility of psychiatric nurses’ professional identity’ (Table 2).

**Table 2:** Nursing managers’ perspectives on factors contributing to abuse.

Theme	Subtheme
Structural challenges embedded in psychiatric care systems	The adverse effects of the healthcare reimbursement system on psychiatric care
	Staffing shortages driven by psychiatric healthcare policies
	The coercive aspects of psychiatric care stemming from treatment limitations
Organizational cultures lacking self-correction	The insularity that discourages external perspectives
	The perpetuation of outdated psychiatric nursing practices
	Reduced sensitivity to inappropriate conduct
	An organizational culture with low barriers to coercion
	A workplace culture lacking in openness
	A workplace culture lacking appreciation and encouragement among staff
Dysfunctional team dynamics that undermine professional competence	Nurse managers who suppress staff autonomy
	A lack of critical reflection in nursing teams
	A lack of collaborative spirit in nursing teams
Asymmetries in patient–nurse relationships	The authoritative nature of psychiatric treatment relationships
	Hierarchical relationships created by the giver–receiver care structure
	The erosion of professional boundaries in patient–nurse relationships
Illness factors that complicate the detection of abuse	Incidences of abuse interpreted as symptoms
	The inability of patients to advocate for themselves
	Patient aggression stemming from persistent symptoms
The fragility of psychiatric nurses’ professional identity	Limited skills and low confidence in psychiatric nurses
	The emotional strain and the sense of being unrewarded in psychiatric nursing
	The erosion of ethical awareness among psychiatric nurses
	The enduring influence of violence in psychiatric nursing
	Individual characteristics of psychiatric nurses

### 3.1 Theme: structural challenges embedded in psychiatric care systems

Nursing managers in this study recognized that abuse against patients was associated with the structure of psychiatric care systems. The participants noted that in the Japanese healthcare system, systemic limitations in mental healthcare, especially regarding finances and staffing, create an environment conducive to abuse.

*“You know, the reimbursement rates for psychiatry are low to begin with, right? So, we try to claim whatever we can. But even then, the rates are still low. And when you’re trying to keep the hospital running smoothly with that, it inevitably leads to certain strains—that’s just the reality.” (Participant 14)*

*“You know, with seclusion, the doctor has to do so many frequent examinations, and with restraints, even more ... But honestly, it’s a joke, isn’t it? Like, are they seriously charging money for this? ... really?” (Participant 14)*

Some participants reported that the adverse influence of the healthcare reimbursement system also led to a deterioration of the ward environment and working conditions. One nursing manager noted that financial constraints within the organization prevented improvements to the facilities, contributing to staff frustration.

*“So hot in our ward. Always complaining, I know, but seriously, everyone’s sweating like crazy—what is this? Patients too. If they don’t fix it, the staff are gonna lose it.” (Participant 7)*

In addition, the staffing standards for psychiatric care in Japan, as established by medical policies, are lower than those for other specialties. The nursing managers in this study expressed concerns that such shortages may contribute to abuse.

*“At my last hospital [a general hospital], it was 1-to-7 staffing, plus eight aides. But here? It’s 1-to-15. Totally different numbers, right? I was shocked by the environment.” (Participant 15)*

*“... short-staffed ... today there aren’t enough nurses, so we have no choice but to use seclusion.” (Participant 10)*

Participants highlighted not only healthcare system limitations but also inherent issues in psychiatry and treatment that contributed to the coercive aspects of psychiatric care.

*“There weren’t many treatment options (at the time), you know. So we had to use force to hold them down to keep everyone safe.” (Participant 10)*

### 3.2 Theme: organizational cultures lacking self-correction

This theme described participants’ views that psychiatric hospitals have an inherent insularity that discourages external perspectives, and the use of outdated psychiatric nursing practices was a barrier to self-correction. Participants highlighted the closed nature of psychiatric hospitals and noted that the prevention of people from outside entering wards and the lack of external oversight may contribute to abuse.

*“If outsiders came into the ward, they’d surely notice if abuse or inappropriate behavior had become normalized.” (Participant 1)*

*“I think the closed-off feeling isn’t good, you know? A space where you can’t feel the gaze of outsiders at all.” (Participant 2)*

In relation to this recognition, one participant felt that the open-door policy in psychiatric hospitals could be an important strategy to prevent abuse. In addition, some of the nursing managers said that the closed environment of the hospital was linked to the practices and beliefs expressed by nurses who had been working in psychiatric hospitals for a long time.

*“Psychiatric hospitals are closed-off, aren’t they? New nurses just follow their seniors ...” (Participant 10)*

*“Older nurses don’t see that their nursing is outdated, and younger ones think it’s good, so the culture keeps getting passed down.” (Participant 16)*

Participants recognized that this type of workplace culture and closed-mindedness reduced sensitivity to inappropriate behavior and lowered the threshold for thoughtlessly imposing behavioral restrictions.

*“I think it’s just something ... used to. The way we interact with patients, especially after being with them for so long, I think.” (Participant 12)*

*“If you don’t address each issue one by one, it becomes habitual, normalized, and leads to major abuse.” (Participant 13)*

*“Each hospital has its own culture and way of thinking, you know ... like, ‘This counts as seclusion’ ... but their values differ ...” (Participant 8)*

Participants believed that characteristics of interpersonal relationships on the wards were linked to abuse. They described a workplace culture lacking in openness and characterized by poor communication, limited exchange of ideas, and a lack of appreciation and encouragement among staff.

*“I think even the staff know it’s not right, but they just can’t say anything. And the people around them feel like there’s no point in speaking up.” (Participant 17)*

*“They don’t want to ruin their relationship by pointing something out, because it might make it harder to work together. I was kind of shocked by that, like, ‘Wait, what?’” (Participant 3)*

*“There’s this atmosphere where staff can’t speak up to their colleagues or supervisors, so they end up having to deal with it on their own. And when they do take it out on someone ... it’s the patients.” (Participant 4)*

### 3.3 Theme: dysfunctional team dynamics that undermine professional competence

This theme highlighted some of the qualities of nursing managers and the collaboration of the nursing team as factors leading to abuse. Participants reported that conservative head nurses removed or suppressed nurses’ autonomy. They perceived that head nurses who suppressed the free opinions and proactive actions of staff were unable to improve the ward culture.

*“People who resist change tend to lean toward being conservative.” (Participant 1)*

*“A manager’s approach—whether they say ‘Let’s try it first’ or focus on risks—makes a big difference. Encouraging staff to try fosters proactivity, while focusing solely on risks can feel dismissive and discourage initiative.” (Participant 1)*

Some participants stated that such a team became less effective at solving problems or collaborating within the group.

*“It seems like they can’t work as a team, whether in medical care or nursing. Maybe they’re just thinking, ‘As long as I’m doing my part, it’s fine.’” (Participant 10)*

*“There’s a lack of team capability—like they don’t have the skills to resolve uncertainties or prepare for the next problem.” (Participant 1)*

In addition, one nurse manager noted that when nurses cannot give each other constructive criticism, they struggle to find solutions other than coercion and control when caring for difficult patients.

*“Teams that only come up with pseudo-solutions in conferences ignore core issues and focus only on what’s in front of them.” (Participant 1)*

*“They only know managerial methods.” (Participant 17)*

### 3.4 Theme: asymmetries in patient–nurse relationships

This theme indicated participants’ belief that asymmetric relationships between patients and nurses were linked to abuse in psychiatric hospitals. Participants described how the coercive nature of psychiatric treatment inherently creates power imbalances, which reinforce the authority of caregivers over patients. This dynamic was seen as a key contributor to abuse.

*“In psychiatry, there’s a lot more need to give guidance or corrections, and that naturally creates a hierarchical relationship.” (Participant 7)*

*“It felt less like they saw the other person as a patient and more like they were just frustrated that the patient wouldn’t listen to them.” (Participant 11)*

Some participants noted that care activities themselves often involve hierarchical relationships because of their inherent giver–receiver nature. This dynamic was particularly evident in tasks like toileting assistance, where caregivers might perceive patients as subordinate.

*“When it’s a ‘giver and receiver’ kind of relationship, the tone of voice tends to become harsher.” (Participant 7)*

*“When it comes to providing care for things like toileting, some people think they have to see the patient as lower than themselves to be able to do it.” (Participant 1)*

*“There are staff who think it’s wrong to apologize to patients.” (Participant 16)*

Participants also noted that in Japanese psychiatric hospitals, the long-term hospitalization of many patients often blurs professional boundaries in patient–nurse relationships, which can lead to abuse.

*“The relationship between patients and nurses seems broken. They’re not family, nor are they beneath them ... (they act that way.)” (Participant 6)*

*“Long-term inpatients. Many staff had worked on the same ward for years. Their ways of addressing or responding to patients were often stricter.” (Participant 9)*

### 3.5 Theme: illness factors that complicate the detection of abuse

Participants reported that factors related to mental illness and patient characteristics, such as symptoms being dismissed or patients being unable to assert their rights, complicate abuse detection in psychiatric hospitals. Some participants noted that although complaints from patients with delusions may be dismissed as “symptoms,” there are instances where some patients report abuse even when no actual abuse has occurred. This further complicated the process of uncovering abuse.

*“When patients say things like, ‘I was hit,’ especially in one-on-one situations, it might be dismissed as part of their delusions or symptoms.” (Participant 1)*

*“(The nurse) didn’t engage in any violent acts, but there are times when patients claim, ‘I was hit’ or ‘I was kicked.’ So, the opposite scenario also happens.” (Participant 1)*

Participants also noted that some patients are bedridden or cognitively impaired and thus unable to assert or defend their rights, which contributes to abuse.

*“In wards with bedridden patients who rarely speak, it’s really challenging.” (Participant 5)*

*“Most of our patients have cognitive impairments, making it hard for them to understand explanations or act on them, so they can’t protect their dignity or advocate for themselves.” (Participant 6)*

Conversely, participants reported that patients whose condition is severe or those who are persistently aggressive place a burden on staff, which contributes to abuse.

*“The patients’ conditions were very severe.” (Participant 17)*

*“When we try to lift behavioral restrictions gradually, their psychiatric symptoms flare up midway and they end up breaking down, hitting staff, over and over again. It’s exhausting for the staff, and you start to feel like giving up.” (Participant 2)*

### 3.6 Theme: the fragility of psychiatric nurses’ professional identity

This theme highlighted the fragility felt by psychiatric nurses about their professional identity, a factor that was often invisible but contributed to abuse. Participants noted that nurses who lacked skills and/or experience had lower confidence. They also noted that emotional strain and the sense of being unrewarded in psychiatric nursing left nurses feeling unfulfilled and exhausted, which may have increased the risk of abuse.

*“(The nurses) have immature knowledge or skills in psychiatry.” (Participant 14)*

*“For example, understanding the patient’s state or why they behave the way they do—those areas of knowledge seem to be somewhat lacking, and I think that has an impact.” (Participant 3)*

One participant stated that the lack of such skills or knowledge led to the use of force to restrain agitated patients.

*“When dealing with psychomotor agitation, it often felt like a force-against-force kind of response.” (Participant 18)*

Some participants reported feeling inferior or servile owing to their role as psychiatric nurses.

*“I feel like psychiatry tends to have an inferiority complex compared to acute care or emergency centers, even though the professional resolve is the same.” (Participant 10)*

*“When I asked, ‘What can you confidently say you’re good at?’ most people answered, ‘Nothing.’” (Participant 15)*

Participants reported that they did not experience satisfaction or a sense of accomplishment in nursing patients, and that the burden of care and exhaustion from frequent complaints sometimes led to frustration being directed toward the patients.

*“The lack of joy or fulfillment in nursing patients is a really big factor.” (Participant 10)*

*“It was such a demanding ward in terms of caregiving, and with the staff shortage on top of that, their stress would end up being taken out on the patients.” (Participant 4)*

Some participants stated that nurses working under daily behavioral restrictions and exposure to violence in environments shaped by structural issues in mental healthcare and organizational culture are more likely to have a weak sense of ethical awareness as professionals.

*“(Between patients) there were often fights or constant small clashes.” (Participant 5)*

*“Seclusion and restraints are heavy-handed treatment measures for patients, but there were staff who treated them as routine and used them as if it were normal.” (Participant 3)*

*“I wonder if it comes down to individual factors—like low ethical sensitivity, complacency, or perhaps a kind of personal weakness.” (Participant 3)*

One participant noted that experiences of violence shaped not only by hospital settings, but also by historical societal norms may lower the threshold for abusive behavior toward patients. As they explained, “it’s tied to that era when physical punishment as discipline was normalized.” Nursing managers in this study also reported that the individual characteristics of nursing staff contributed to factors associated with abuse.

*“I think it probably has something to do with how that person was raised. But there are things they just can’t stop themselves from doing.” (Participant 16)*

*“Certain biases in approach, which could lead to inappropriate actions, may sometimes be linked to individual traits, including developmental challenges. This isn’t limited to psychiatric nursing; it can happen in any area of nursing.” (Participant 18)*

## 4 Discussion

This study is one of the first to clarify the factors contributing to abuse in psychiatric hospitals from the perspective of nurse managers. The identified themes encompass a wide range of characteristics, including not only nurse-related factors but also organizational and ward cultures, as well as structural issues inherent in psychiatric care systems.

The abuse of people with mental illness has been previously discussed in relation to coercive practices and the hospital ward environment (Ntsaba and Havenga, 2007; Wood and Pistrang, 2004). In the present study, nursing managers recognized the mental healthcare system and structure as indirect contributors to abuse in psychiatric hospitals. Participants reported that low staffing levels lead to understaffing, which results in inadequate patient care and the casual use of coercive measures. Low nurse-to-patient ratios contribute to a decline in the quality of care (Kohanová et al., 2024; Mitikhin et al., 2024), and it has been suggested that nursing shortages and the absence of physicians may lead to the use of coercive practices (Schreiber et al., 2022). Previous research exploring the experiences of patients subjected to seclusion and restraint has also highlighted concerns regarding insufficient staff support, including non-responsiveness to patients' requests for assistance, contributing to an inappropriate and neglectful care environment (Ntsaba and Havenga, 2007). Additionally, low staffing levels have been associated with nurse fatigue (Ohnishi et al., 2010), and nurses experiencing fatigue or burnout tendencies may be more likely to engage in inappropriate conduct (Austin et al., 2003; Epstein et al., 2023). A qualitative study conducted in Canada investigating the experiences of psychiatric nurses suggested that insufficient staffing and time constraints led to treating patients as “objects,” which caused moral distress among nurses (Austin et al., 2003). Therefore, the present findings suggest the need to develop policies that ensure adequate staffing in psychiatric care to prevent abuse of patients in psychiatric hospitals.

Psychiatric nurses experience moral distress when they have to provide inadequate care because of understaffing, tolerate unethical behaviors of colleagues, or overlook violations of patient rights (Corley et al., 2005; Ohnishi et al., 2010). In this study, nurse managers recognized situations that elicit moral distress as factors linked to organizational and ward cultures, which they perceived as contributing to abuse. In particular, tolerating the unethical behavior of colleagues and overlooking violations of patient rights were identified as subthemes in the Organizational cultures lacking self-correction theme. These subthemes included a closed-off workplace culture and the perpetuation of outdated psychiatric nursing practices. These organizational culture aspects were also related to the dysfunctional team dynamics that undermine professional competence. These findings suggest that combined with inadequate leadership, organizational cultures that lack self-correction create an environment where abuse is more likely to occur. Furthermore, the findings highlight the fragility of psychiatric nurses’ professional identity, a factor characterized by limited skills, low confidence, and the erosion of ethical awareness. These factors, combined with the emotional strain and moral distress experienced by nurses, create conditions in which inappropriate behavior may become normalized, thereby increasing the risk of abuse (Lamoureux et al., 2024). The attitudes and leadership of nurse managers were considered critical factors that influenced ward culture and team dynamics. Previous studies have reported that poor-quality leadership is associated with environments where abuse occurs (McGarry, 2019), and nurse managers who suppress staff autonomy may create conditions conducive to abuse. Nurse managers play a pivotal role in fostering a supportive ward environment and ensuring the practice of patient-centered care (Beckett et al., 2013; Teixeira et al., 2022). Therefore, developing effective nurse managers is essential to prevent abuse and create safer psychiatric wards.

The insularity of psychiatric hospitals, which limits external oversight, was also identified as a factor contributing to abuse. Although research on the factors contributing to abuse in psychiatric hospitals remains limited, studies on elder care facilities have reported that patients who do not receive regular visits are more likely to experience lower-quality care or abuse (Hsieh et al., 2009; Joshi and Flaherty, 2005). Similar situations exist in psychiatric wards, where a lack of external contact creates a closed-off environment. Therefore, increasing openness in psychiatric hospitals may be important in preventing abuse. A quasi-experimental study in psychiatric hospitals demonstrated that implementing open-door policies did not increase adverse events such as violence, but reduced the use of coercive treatments. This suggests that open-door policies can be implemented without exposing patients to undue risks (Schreiber et al., 2022). Participants in the present study also recommended increasing openness in psychiatric hospitals as a measure to prevent abuse. In a previous study, nurses working in psychiatric acute wards with open-door policies reported that their practices, including “real respect for the person,” “ensuring patients’ rights,” and “listening to the person,” fostered trust-based therapeutic relationships and contributed to their own empowerment (Missouridou et al., 2021).

In psychiatric nursing, developing equitable patient–nurse relationships is essential for patient recovery (Matoba et al., 2023). However, an imbalance in the patient–nurse relationship was recognized as a factor contributing to abuse. The inherently coercive nature of psychiatric treatment often imbues the therapist with authority, causing patients to feel belittled by nurses and experience distress and disappointment (Sambrano and Cox, 2013; Yosep et al., 2021). Moreover, the authority of nurses and the absence of an alliance between patients and nurses have previously been noted as barriers to establishing equitable relationships (Khoury, 2019; Sandhu et al., 2015). Therefore, the adoption of recovery-oriented practices in psychiatric wards may be an effective measure for preventing abuse (Lorien et al., 2020; Waldemar et al., 2019).

Patient-related factors such as the inability to voice concerns and symptoms that are concealed as part of the illness were also identified as contributors to abuse. A study that interviewed nurses from psychiatric hospitals where abuse occurred reported that nurses had divided opinions on whether to believe the claims of patients or the alleged perpetrators, owing to the subtle and covert nature of the abuse (McGarry, 2019). Similarly, a qualitative study exploring the experiences of mental health service users identified several reasons why abuse often goes unreported, including users being unable to perceive or articulate their experiences because of their mental state and the fear of not being believed (Carr et al., 2019). One participant in the present study was worried that patients’ concerns are sometimes dismissed as “delusions.” Conversely, a factor that complicates the detection of abuse is the possibility of patients falsely accusing staff of abuse when no such incidents occurred. In such dilemmas, it is essential to establish organizational protocols in advance to respond to allegations of abuse. These protocols should consider both the protection and care of patients who report abuse and the protection and care of staff in cases of false accusations. Integrating a trauma-informed care approach into these responses is particularly important to ensure that all parties are supported appropriately (Carr et al., 2023).

## 5 Limitations

This study had several limitations. First, the findings may not be fully applicable to other contexts. In Japan, long-term hospitalization and the number of psychiatric beds per population are considerably higher compared with other countries (Okayama et al., 2020). However, harmful experiences, including coercive practices in psychiatric hospitals, have also been reported in Western countries (Askew et al., 2019), suggesting the potential for transferability. Further research is needed to investigate these factors in different contexts. Second, the study aim was to elucidate the factors contributing to abuse in psychiatric hospitals from the perspective of nurse managers. It is possible that different factors may emerge from the perspectives of ward nursing staff or patients. Third, the use of a convenience sampling method to recruit participants may have introduced selection bias into the data. To deepen the understanding of factors contributing to abuse, future studies should extend this research by including patients as participants and using quantitative methods.

## 6 Conclusions

We conducted thematic analysis to elucidate the factors contributing to abuse of inpatients in psychiatric hospitals, as perceived by nurse managers. Psychiatric nurse managers recognized that factors such as the personal traits of nurses, the dynamics of nursing teams, and the characteristics of patients’ illnesses were associated with abuse. Additionally, the structure of the mental healthcare system and the organizational culture of psychiatric hospitals in Japan may contribute to the occurrence of abuse. These findings suggest that preventing abuse in psychiatric hospitals requires a comprehensive organizational strategy that includes staff education, leadership training, and fostering a culture of self-correction within teams. Future research should aim to develop and evaluate effective prevention strategies that incorporate perspectives from patients and ward staff to ensure a holistic understanding and robust interventions against abuse.

## Glossary

Credibility: the interpretation of data closely represents the participants’ experiences.

Reflexive thematic analysis: An interpretative approach to qualitative data analysis that involves active engagement with the data and permits the dynamic identification and analysis of themes based on emergent patterns.

Trustworthiness: the data analysis has been conducted in a comprehensive, consistent, and accurate manner.

Dependability: the qualitative research process is traceable, logical, and clearly recorded.

Transferability: the extent to which the findings can be generalized to other contexts.

## CRediT authorship contribution statement

**Kei Matoba:** Formal analysis, Investigation, Project administration, Validation, Writing – original draft, Conceptualization, Data curation, Funding acquisition, Methodology, Resources, Visualization, Writing – review & editing. **So Yayama:** Validation, Formal analysis, Resources, Investigation, Writing – review & editing. **Taiki Teshima:** Validation, Formal analysis, Writing – review & editing. **Akiko Miki:** Validation, Formal analysis, Writing – review & editing.

## Declaration of competing interest

The authors declare the following financial interests/personal relationships which may be considered as potential competing interests:

Kei Matoba reports financial support was provided by Japan Society for the Promotion of Science. Kei Matoba reports a relationship with GLOME Worksupport, INC. that includes speaking and lecture fees. If there are other authors, they declare that they have no known competing financial interests or personal relationships that could have appeared to influence the work reported in this paper.

## Appendix Supplementary materials

Image, application 1
